# Special Issue “Gut Microbiota, Inflammatory Bowel Diseases, and Therapeutic Targets”

**DOI:** 10.3390/ph16050714

**Published:** 2023-05-08

**Authors:** Eirini Filidou, George Kolios

**Affiliations:** Laboratory of Pharmacology, Faculty of Medicine, Democritus University of Thrace, Dragana, 68100 Alexandroupolis, Greece; efilidou@hotmail.com

The gut microbiota and its overall genetic composition, the microbiome, have been the subject of extensive research over the last decade within the fields of genomics, transcriptomics and metabolomics, and their role in various other targeted approaches and advanced technologies has been explored [1]. Various studies have demonstrated that the composition of the gut microbiota differs in patients with Inflammatory Bowel Disease (IBD) compared to the eubiotic microbiota of healthy controls [2]. Additionally, gut microbiota is different between patients with Ulcerative Colitis (UC) and Crohn’s Disease (CD) or among subtypes of CD [3]. This dysbiotic composition involves an increase or decrease in specific intestinal bacterial species in patients with IBD, and more specifically, a decrease in the abundance and diversity of specific genera has been suggested to play a key role in the pathogenetic mechanisms of intestinal inflammation [4]. Having underlined the significant role of gut microbiota in IBD, this Special Issue was mainly focused on its involvement in health and IBD pathogenesis, its interactions and effects on the host, and on new possible therapeutic strategies involving the use of probiotics.

Most studies in IBD have been focused on microbiome disturbances in the lower gastrointestinal tract, where bacteria are more abundant, and a dysbiosis of gut microbiota has been documented, whereas similar studies in the duodenum are rare, mainly in animal models [5,6]. In this Special Issue, Schmidt et al. studied the composition of the bacterial flora, attached to the duodenal mucosa, in children with CD, since the prevalence of CD is more common in children [7]. They examined bacterial relative abundance, alpha and beta composition, and diversity, analyzing the 16S ribosomal RNA bacterial gene in biopsies from duodenum and terminal ileum from treatment-naïve CD children and age- and sex-matched controls. They demonstrated that the duodenal microbiome in children is distinct from the terminal ileum and is characterized by an increased abundance of Bacteroidales in active CD children and by an increased abundance of Pseudomonodales and Spirochetes in healthy controls.

In addition to changes in microbial diversity in IBD, distinct microbial signatures have been reported in subtypes of the disease or in cases of colectomy or complications [8]. Dovrolis et al. have shown distinct microbiome signatures in complicated Crohn’s disease subphenotypes, with the bacterial diversity of the inflamed group found to be closer to that of healthy controls than to that of structuring and penetrating groups [3]. In this Special Issue, Bálint et al. examined the possible effect of anatomical variations, after colectomy, on the diversity of gut microbiota, using a clinical model of two separate bowel conditions: UC patients after ileal pouch-anal anastomosis (IPAA) surgery, compared with UC patients, familial adenomatous polyposis (FAP) patients after IPAA surgery and healthy controls [9]. They found that the microbiota diversity of the non-IBD colectomized group was closer to that of the colectomized UC patients than the healthy controls, suggesting that the anatomical status construct has an influence on microbiota composition, in addition to intestinal inflammation.

The microbiota metabolism ends in the production of several metabolites that participate in interactions with the host and ultimately regulate mucosal homeostasis in gut and influence the pathophysiology of various disorders [10,11]. In addition, the interaction between the intestinal microbiota and the host at the metabolite receptor level appears to be involved in intestinal inflammation and IBD [12]. Ιn this Special Issue, three reviews summarize the recent knowledge and discuss in detail the role of the microbial metabolites in IBD. A comprehensive review by Aldars-García et al. studies the implication of microbial metabolites in the pathogenetic mechanisms of IBD. Furthermore, it examines the changes in the metabolome related to disease subphenotype and response to treatment, as well as the possibility that these changes may be useful biomarkers in the management of patients with IBD [10]. Another review, by Dowdell and Colgan, gathers the current knowledge on metabolic host–microbiota interactions in the pathogenesis of IBD, focusing mainly on their implication in autophagy and their role in gut inflammation [13]. Finally, Bunt et al. comprehensively examine various metabolites produced through the fermentation and metabolism of various dietary sources by gut microbiota and report data showing anti-inflammatory and antioxidant properties of these metabolites in in vitro and in vivo models of IBD [14]. They also discuss the potential therapeutic effects of these compounds. Existing studies have revealed a critical role played by microbial metabolites as effectors for productive innate immunity and maintenance of homeostasis, and their changes are associated with mechanisms of intestinal inflammation. However, at present, there is insufficient evidence to determine the role of the microbial metabolite–host receptor interactions in pathogenesis and/or the potential for translating it into clinical practice.

The central role of the gut microbiota in regulating health and disease, mentioned earlier, has received prominent emphasis in the development of potential therapeutic strategies. In the regulation of gut microbiota and treatment of dysbiosis, various methods such as fecal transplantation and probiotic, prebiotic and postbiotic approaches have been used to influence the host microbiota and treat IBD [15,16,17,18]. It appears that effective modulation of the microbiota composition may be a therapeutic approach or may enhance the effectiveness of an existing treatment [19]. In this Special Issue, Filidou & Kolios present and discuss the current knowledge of the mechanisms underlying the healing process in the intestinal mucosa and examine the role of the gut microbiota in healing pathophysiological mechanisms in gut homeostasis and intestinal inflammation [20]. They also discuss the steps in these host–microbe interactions that result in mucosal healing, highlighting possible therapeutic targets, presenting current data on the effect of probiotics on intestinal healing in animal models of colitis, and indicating that probiotics can even lead to the amelioration of intestinal inflammation and improvement in mucosal wound healing. 

Tarapatzi et al. in vitro examined the role of certain probiotics strains in wound healing, using cultures of subepithelial gut myofibroblasts; in this Special Issue, the authors demonstrated that the mix of Bifidobacterium lactis, Lactobacillus acidophilus, Lactiplantibacillus plantarum and Saccharomyces boulardii induced a moderate, but statistically significant, increase in the mRNA of certain healing factors, such as collagen type I and III, fibronectin and tissue factor and myofibroblasts migration, thus indicating a positive effect in wound healing [21]. In addition, they found that this probiotic combination induced a mild increase in the mRNA of specific chemokines, and they suggested that this was due rather to a possible immune alertness than a pathological inflammatory response, contributing in this way to the host’s defenses. The use of multiple probiotic strains was found to have a better effect on mucosal immunity than the use of single probiotics. 

It has also been suggested that the regulation of microbiota composition may enhance the effectiveness of an existing treatment [19]. In this Special Issue, Saber et al. examined a possible synergistic effect of rosuvastatin and Lactobacillus in an experimental model of colitis [22]. Previous studies have shown a protective effect of rosuvastatin in animal models of colitis, and it has been suggested as a potential therapeutic agent in IBD [23,24]. In this study, the combination of rosuvastatin/Lactobacillus was found to be safe and decreased the Disease Activity Index of colitic animals via an anti-inflammatory effect, thus indicating a possible role in the management of IBD.

In conclusion, this Special Issue has once again highlighted that in IBD, the gut microbiome is distinct and varies from healthy controls, and that the anatomical variations in the intestine can significantly influence its composition. Apart from the microbiome itself, it is also underlined that a dysbiotic microbial metabolome may also contribute to the IBD pathogenesis, while some may exert positive effects on the host’s immune functions. Finally, aiming in this direction, this Special Issue highlights the potentially beneficial role of probiotics, not only in mucosal healing, but also in triggering and maintaining a mild immunological alertness in the host’s cell populations.

## Data Availability

Not applicable.
